# Serious and Fatal Illness Associated with Falciparum and Vivax Malaria among Patients Admitted to Hospital at West Sumba in Eastern Indonesia

**DOI:** 10.4269/ajtmh.2012.11-0577

**Published:** 2012-07-01

**Authors:** Siti Nurleila, Din Syafruddin, Iqbal R. F. Elyazar, J. Kevin Baird

**Affiliations:** Eijkman-Oxford Clinical Research Unit, Jakarta, Indonesia; Eijkman Institute for Molecular Biology, Jakarta, Indonesia; the Centre for Tropical Medicine, Nuffield Department of Medicine, Oxford University, Oxford, United Kingdom

## Abstract

Records of 3,449 patients admitted to Karitas Hospital at Waitabula in eastern Indonesia with microscopy-confirmed malaria through 2008 and 2009 were systematically reviewed. Falciparum, vivax, and mixed species malaria occurred among 1,541, 1,837, and 71 admissions, respectively. Among these, 400 (26%), 199 (11%), and 15 (21%) had serious illness. Fatalities occurred in 46 (12%), 18 (9%), and 2 (13%) of these patients, respectively. Although patients with a diagnosis of falciparum malaria were more likely to have serious illness compared with those with vivax malaria (odds ratio [OR] = 2.9; 95% confidence interval [CI]: 2.4–3.5), this diagnosis nonetheless was associated with 32% of serious illness and 27% of fatalities. Among the seriously ill with a diagnosis of falciparum or vivax malaria, no significant difference in risk of death occurred (OR = 1.3; 95% CI: 0.7–2.5). Serious and fatal illness was predominantly anemia or altered mental state syndromes among patients with either of the species diagnoses. *Plasmodium vivax* was associated with a substantial share of the burden of morbidity and mortality caused by malaria in this hypo- to meso-endemic community.

## Introduction

*Plasmodium vivax* malaria has long been considered only rarely fatal.^1^ Recent studies, however, have revitalized the argument that *P. vivax* may often become pernicious and directly threaten life.^2^ As the causal agent in 100–400 million clinical attacks each year,^3^ and threatening almost three billion people exposed to endemic risk,^4^ this parasite obviously bears consideration as a major global health concern.

Recent work from India emphasizes the uncertainty in estimates of mortality caused by malaria. One study used verbal autopsy methods and the findings suggested underestimation of the burden of malaria mortality in that nation by at least 9- and as much as 19-fold^5^; given the broad presumption of the benign character of *P. vivax*, perhaps few would credit even a minor share of mortality attributed to malaria to this parasite. Nonetheless, recent retrospective and prospective hospital-based studies^6^–^14^ involving 18,141 patients, 13% and 14% were classified as having serious illness with a diagnosis of *Plasmodium falciparum* or *P. vivax*, with 10% and 7% of those ending in death, respectively. Such evaluations, comparing relative burdens associated with the two species, may mitigate some of the many confounding factors (misdiagnosis, concurrent infections, and underlying disease) inherent to endemic zones like Indonesia.

In 2006 Indonesia reported 347,597 confirmed and suspected malaria cases to the World Health Organization (WHO), most of those having a laboratory-confirmed diagnosis.^15^ The World Health Organization estimated 2.5 million clinical attacks in Indonesia for that year. According to Malaria Atlas Project (MAP), the annual number of clinical attacks of *P. falciparum* alone was over 12 million, and the number of Indonesians at risk of *P. falciparum* in 2007 was 151 million,^16^ whereas those at risk of *P. vivax* in 2009 numbered 175 million.^3^ The number of clinical attacks of vivax malaria among Indonesians has not yet been reliably estimated, but historically accounts for 46% of prevalent malaria in blood screening surveys.^15^ Thus, malaria represents a very substantial threat to public health in Indonesia, and vivax malaria constitutes a considerable contribution to that broader problem.

Most endemic malaria in Indonesia is relatively low transmission intensity^16^; to better grasp the relative contributions of falciparum and vivax malaria to associated morbidity and mortality in this setting, a retrospective analysis of admissions to a single hospital in West Sumba, eastern Indonesia, over 2008 and 2009 was conducted.

## Materials and Methods

### Study site.

Karitas Hospital is located at Waitabula near the northern coast airport of Tambolaka in West Sumba, Indonesia. Karitas serves as the primary hospital in that community, and referral site for health centers throughout much of West Sumba. The catchment population was ∼280,000 people. Karitas had 115 beds for inpatient care, surgical capacities, and a clinical laboratory service. People coming to the hospital seeking treatment of febrile illness first had blood smears examined for malaria by microscopy. Technicians trained and certified as competent both by the Eijkman Institute for Molecular Biology and U.S. Naval Medical Research Unit 2^17^ in Jakarta read the blood smears. Once diagnosed with malaria, patients were referred to a physician for evaluation. They were managed either as an outpatient or admitted for treatment and observation as a malaria inpatient. The hospital maintained records of diagnosis and treatment of all of these outpatients and inpatients.

A systematic sampling of villages in West Sumba in 2007 for cross-sectional blood surveys of 8,870 residents of 45 villages showed wet and dry season prevalence for *P. falciparum* of 4.88% and 2.88%, respectively, and 2.16% and 2.14% for *P. vivax*.^18^ Prevalence of malaria among villages ranged from 0% to 34%, however with only four of the 45 villages sampled having prevalence exceed 20% at either wet or dry season samples.^18^ Endemic malaria in West Sumba thus typically occurs at a prevalence of < 5% with relatively little difference between *P. falciparum* and *P. vivax*.

### Patient records management.

The ethical review boards of the Eijkman Institute for Molecular Biology and of the London School of Hygiene and Tropical Medicine reviewed the protocol describing this study and approved its conduct. This retrospective, cross-sectional study retrieved the records of patients who sought treatment at Karitas Hospital and who had a laboratory-confirmed malaria diagnosis.

**Figure 1.**
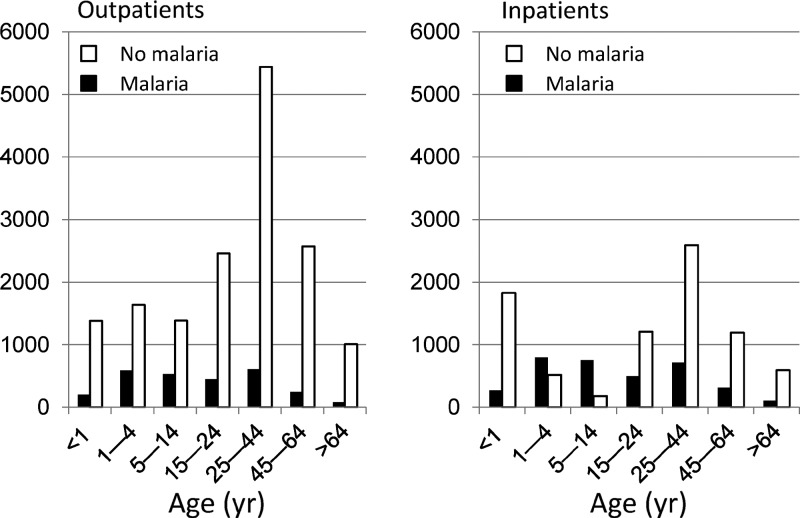
Graphs illustrate the number of outpatients (left) and inpatients (right) among demographic groups at Karitas Hospital, West Sumba, Indonesia during 2008 and 2009.

Outpatient and inpatient records for all of 2008 and 2009 were examined. Figure 1 illustrates the numbers of patients with a microscopically confirmed diagnosis of malaria across demographic groups for both inpatients and outpatients. Among 30,142 hospital visits, 2,711 (9%) were confirmed positive for malaria and managed as outpatients. Another 3,484 (11%) were admitted as inpatients; 35 of those records were excluded from analysis because of incomplete or illegible content, and thus 3,449 inpatient records constituted the study population.

Relevant data from the hospital patient file, with exclusion of personal identifiers, was directly transferred into two duplicate case report forms (CRF) for each patient by two different nurse technicians. Each CRF was then manually entered into an electronic database (EpiData) twice. The four electronic versions per patient were examined for discrepancies that were resolved by manual cross-check of the CRF copies and, if necessary, the hospital patient record. Concordant electronic CRFs were uploaded to a permanent storage device in Stata version 11 (Stata, College Station, TX) for analysis.

### Classification of patients.

Infants were < 12 months of age, children were < 10 years of age, adolescents were between 10 and 15 years of age, adults were > 15 years of age, and people > 60 years of age were classified as elderly. Women were classified as pregnant with affirmative evidence in the record; absent of such evidence they were classified as not pregnant. The malaria microscopists did not count and record parasite densities from blood films. Instead they used a scoring system based on the number of parasites seen among microscopic fields of the thick film under oil immersion magnification (1,000×) as follows: 1+ = 1–10 parasites/100 fields, 2+ = 11–100 parasites/100 fields, 3+ = 1–10 parasites/field, and 4+ ≥ 10 parasites/field. The 4+ score may be estimated at > 6,000/μL by assuming that each high power field represents ∼1/600th of a microliter.^19^ Although scores were available for almost all patient records, 6,000 parasites/μL was too low a threshold for classifying patients as being seriously ill on the basis of relatively heavy parasitemia. The parameter was applied to examine the frequencies of relatively high parasitemia between the two species among patients classified as not serious, serious, or fatal.

Classification of disease severity did not comply with standard definitions such as those provided by WHO because most measurements required for that definition were not available at Karitas Hospital. Strict application of it would have caused exclusion of most records as incomplete. Therefore, before examining the records, a system for classification of illness associated with a diagnosis of malaria was developed using what was understood to be broadly available information at Karitas.

Patients meeting the following criteria were classified as having NOT SERIOUS illness:

1. Not admitted to intensive care unit; and
2. No record of seizure or altered consciousness; and
3. No record of severe anemia (< 5 or < 7 g/dL for children or adults) or transfusion; and
4. No record indicating pulmonary distress; and
5. No record indicating renal failure; and
6. No record indicating hepatic failure.

Patients were classified as having SERIOUS illness in accordance with the following:

1. Treated in any ward; and
2. Intravenous (IV) antimalarial therapy; or
3. Record of seizure or altered consciousness; or
4. Record of severe anemia (< 5 and < 7 g/dL for children or adults) or transfusion; or
5. Record indicating pulmonary distress; or
6. Record indicating renal failure; or
7. Record indicating hepatic failure.

Patients classified as having SERIOUS malaria were further classified according to the apparent organ of primary illness. All of these classifications were made on the basis of notes or data in the patient record. The following guided these syndromic classifications:

1. Severe anemia: Patient record contained notes documenting blood transfusion or a laboratory measurement of hemoglobin < 5 g/dL for children and < 7 g/dL for adults.
2. Altered mental state: Patient record contained notes documenting patient was disoriented, stuporous, delirious, unconscious, comatose, suffering at least two seizures, and receiving intravenous or intramuscular antimalarial therapy.
3. Respiratory distress: Patient record contained notes describing labored or rapid breathing (> 50 breaths/minute).
4. Hepatic illness: Patient record contained notes describing physical signs of jaundice or containing record of laboratory values aspartate aminotransferase (SGOT) and alanine aminotransferase (SGPT) elevated 2.5-fold above normal values (0–40; 0–50).
5. Bleeding: Patient record contained notes describing patient had spontaneous bleeding from any site.
6. Prostration: Patient record contained notes describing patient was unable to sit up or stand without assistance.
7. Double syndrome: Any two of the above.
8. Multiple syndromes: More than two of the above.

Patients were classified as FATAL only with evidence that death occurred in the hospital and the attending physician recorded malaria as the cause of death.

### Statistical analysis.

The statistical analysis used Stata version 11. Descriptive analyses were performed to determine the proportions for categorical data. Odds ratios (OR), 95% confidence interval (CI), and *P* values comparing rates and proportions were calculated using χ^2^ contingency tables and Fisher's exact test. The CI that included 1 and/or the *P* values that were > 0.05 were considered insignificant.

## Results

**Figure 2.**
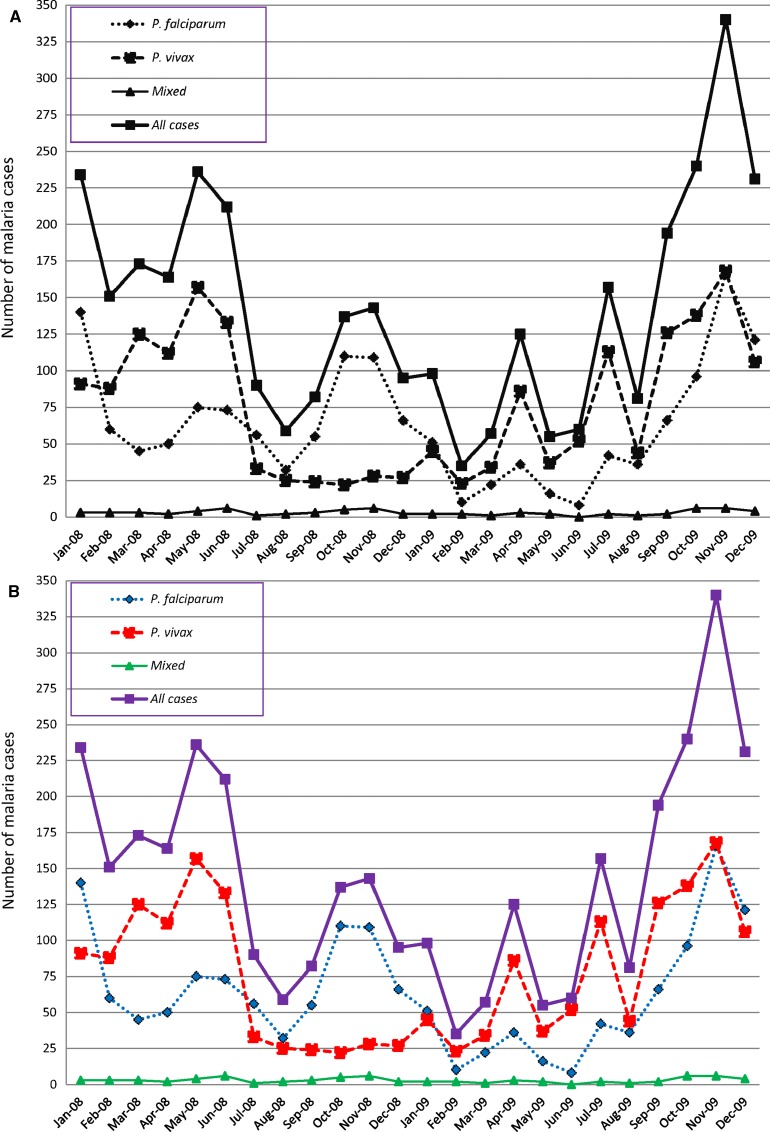
Graph illustrates monthly numbers of admissions to Karitas Hospital in West Sumba, eastern Indonesia, ascertained by a retrospective review of admission records. The lines show all admissions (X, purple), falciparum malaria (diamond, blue), vivax malaria (square, red), and mixed infections of these two species (triangle, green).

Figure 2 illustrates monthly admissions to the hospital for malaria over the 24 months of analysis by species of diagnosis. Table 1 lists the distribution of admissions, serious illness, and death with a diagnosis of *P. falciparum*, *P. vivax*, or mixed infections of these two species among demographic groups. Among the 3,449 patients admitted with a diagnosis of malaria, 614 (18%) were classified as having serious disease, and 66 (11%) of those patients died. Falciparum malaria occurred among 45% of admissions, 65% of serious disease, and 70% of deaths, whereas vivax malaria occurred in 53%, 32%, and 27% of these, respectively. No significant differences appeared between rates of serious illness with a diagnosis of *P. falciparum* versus mixed species infection (26% versus 23%; *P* = 0.527), and the same was true of case fatality rates (12% versus 13%; *P* = 0.905). Mixed infections were excluded from further analyses comparing trends in morbidity and mortality between species.

Presumptive antibiotic therapy was not routinely administered with a diagnosis of malaria. Among the 220 patient files randomly selected for the survey of antibiotic therapy (110 *P. falciparum* and 110 *P. vivax*), 85% and 72% received no antibiotics, respectively. Antibiotic therapy was with amoxicillin, ampicillin, or ciprofloxacin.

Table 2 lists ORs for risk of serious illness with a diagnosis of *P. falciparum* relative to *P. vivax* among demographic groups. Overall, patients admitted with a diagnosis of falciparum malaria were 2.9 times (95% CI: 2.4–3.5) more likely to be classified as having serious illness compared with admission with a diagnosis of vivax malaria. This trend was strongest among infants, children, and adolescents (OR 3.0 to 8.3), and weakest among adults and the elderly (OR 1.3 and 0.4). Pregnant women were no more likely to have serious illness with a diagnosis of falciparum compared with vivax malaria (OR = 0.7; 95% CI: 0.3–2.1), and this was also true among women classified as not pregnant (OR = 1.4; 95% CI: 0.8–2.4). Compared with not pregnant women, pregnant women with a diagnosis of falciparum malaria or of vivax malaria were similarly likely to be classified as having serious disease (OR = 0.9; 95% CI: 0.4–2.1, and OR = 0.5; 95% CI: 0.2–1.2, respectively).

**Figure 3.**
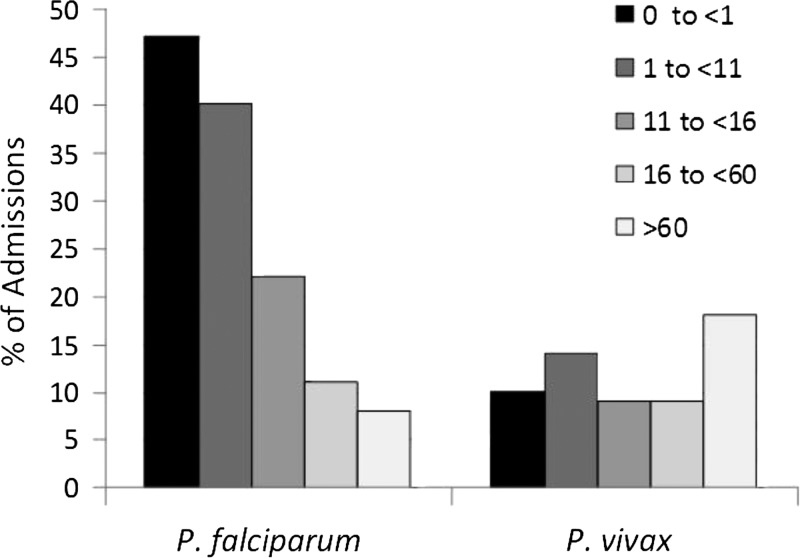
Age-dependent diminishing risk of being classified as having serious illness among patients admitted with a diagnosis of falciparum malaria, and absence of the same with a diagnosis of vivax malaria. Legend lists groups according to year of age.

Infants, children, and adolescents contributed 82% of serious illness with a diagnosis of *P. falciparum* and 54% of that for *P. vivax*. Overall, these young age groups accounted for 73% of serious illness and 77% of fatalities, and represented only 55% of the general population. They were 4.5 times (95% CI: 3.3–6.1) more likely to have serious outcomes with a diagnosis of *P. falciparum* compared with adults and the elderly with the same diagnosis. The same was not true for a diagnosis of *P. vivax*, where risk of serious illness appeared more evenly distributed between infants/children/adolescents versus adults/elderly (OR = 1.2; 95% CI: 0.8–1.7). Figure 3

illustrates the distinct age-related susceptibility to serious illness with a diagnosis of falciparum malaria among admitted patients, and the absence of such an effect with a diagnosis of vivax malaria. Table 3 lists ORs for fatal outcomes with serious falciparum- versus vivax-associated illness. The odds of death among seriously ill patients were no different between diagnoses of the two species (OR = 1.3; 95% CI: 0.7–2.5).

**Figure 4.**
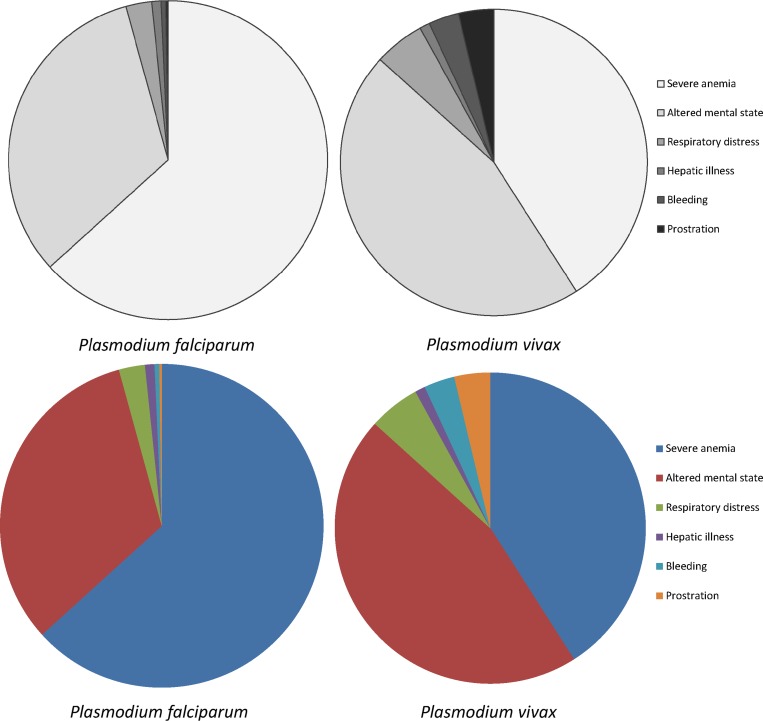
Charts illustrate the relative frequencies of syndromes among patients classified as having serious illness with a diagnosis of falciparum or vivax malaria.

Figure 4 illustrates the distribution of syndromes among all patients having falciparum or vivax malaria. Among 400 patients with a diagnosis of falciparum malaria and classified as having serious illness, severe anemia (266) and altered mental state (137) overwhelmingly dominated among other possible syndrome classifications. The pattern was similar among patients with vivax malaria; severe anemia (77) and altered mental state (86) were dominant among the 199 patients. The majority of patients with severe anemia received transfusions; 92% and 84% for falciparum and vivax malaria patients, respectively. Other syndromes associated with falciparum and vivax malaria were respiratory distress – 11 and 10; hepatic illness – 4 and 2; bleeding – 2 and 6; and prostration – 1 and 7, respectively. Syndromes among the few serious mixed infections (15) were essentially similar to those for falciparum and vivax malaria. Significant differences in the relative frequencies of syndromes between children and adults appeared only with altered mental state in vivax malaria (55% and 29%, respectively, *P* = 0.02). Most patients with serious illness and a diagnosis of falciparum (90%) or vivax (96%) malaria presented with only a single syndrome, and only one patient presented with more than two syndromes (Table 4). Most of the fatal cases with a diagnosis of vivax malaria showed only a single syndrome (16 of 18), and this was also true of fatalities associated with falciparum malaria (36 of 46).

**Figure 5.**
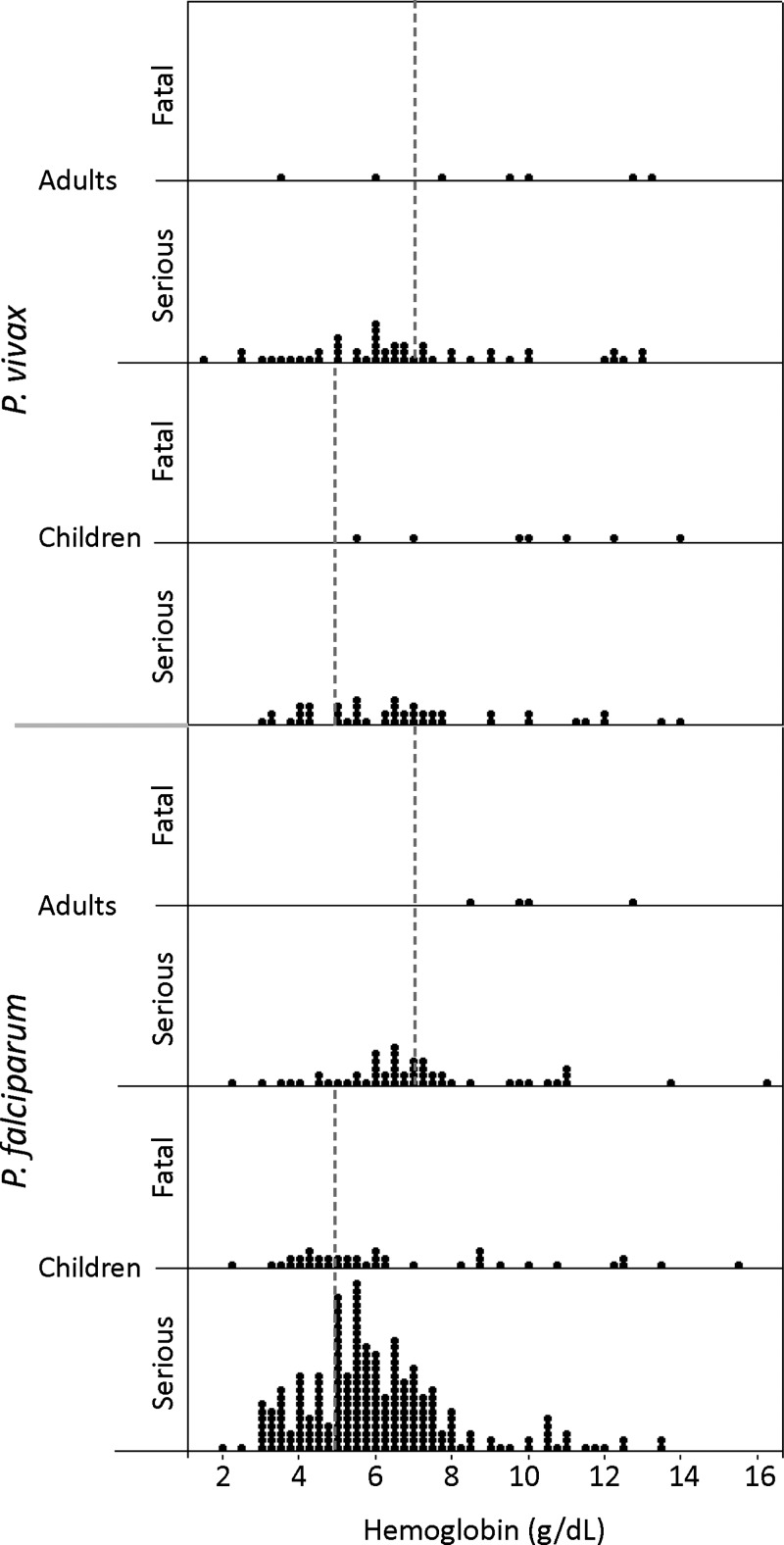
Distribution of available hemoglobin measurements among patients classified as having serious illness by any syndrome. The hashed vertical line represents the thresholds for severe anemia among adults and children.

Figure 5 illustrates available hemoglobin measurements among seriously ill and fatal cases (any syndrome). The distribution of these measurements around the respective thresholds for severe anemia was similar across species of diagnosis, demographic group, and clinical severity of disease. One possible exception was the absence of severe anemia among fatal cases with a diagnosis of *P. vivax* in children, but the few cases did not permit adequate statistical inference on this distribution.

**Figure 6.**
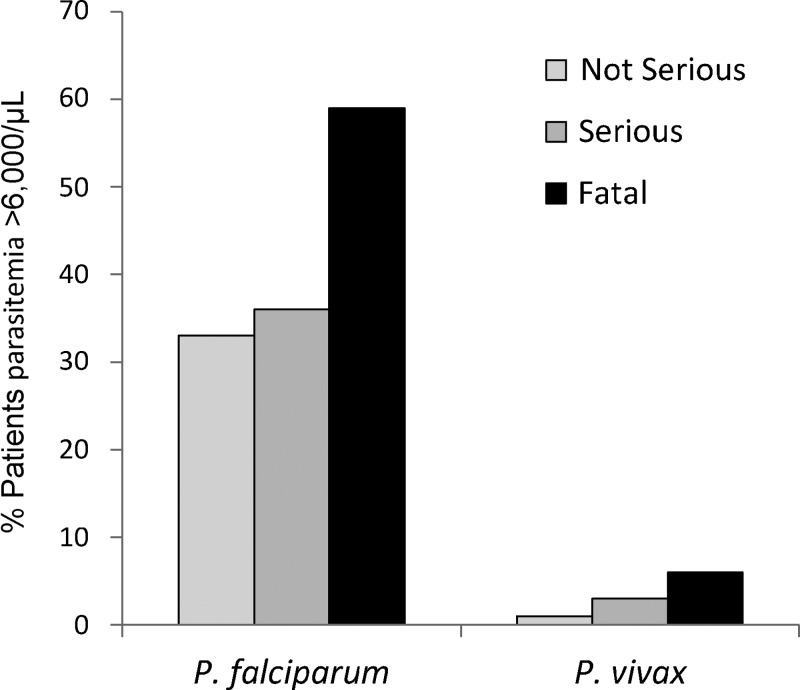
Frequency of parasitemias exceeding ∼6,000 parasites/mL among patients with not serious, serious, and fatal infections by *Plasmodium falciparum* or *Plasmodium vivax*.

Figure 6 illustrates the distribution of parasite counts scored 4+ and therefore higher than ∼6,000 parasites/mL blood among not serious, serious, and fatal disease associated with falciparum or vivax malaria. Many patients with serious and fatal falciparum malaria had relatively high parasitemias (33–59%) and the proportion having > 6,000/μL increased with increasingly serious illness. In contrast, a minority of patients with vivax malaria had high parasitemia scores (< 6%), and these relatively very low frequencies increased with increasingly serious illness (0.6–6%).

## Discussion

This study analyzed trends in morbidity and mortality among patients admitted to a tertiary care referral hospital at West Sumba in eastern Indonesia with a microscopic diagnosis of malaria during 2008 and 2009. The relatively compact and well-resourced Karitas Hospital provided access to remarkably thorough and complete patient files, coupled with systematic and certifiably competent microscopic diagnoses of malaria. Like most retrospective hospital-based analyses of illness associated with malaria, this one applied an improvised and simplified definition of serious illness with malaria. Although this requirement imposes uncertainty with respect to comparability of the risk findings with those in other hospitals, the uniform application of this definition to patients at Karitas Hospital nonetheless provides useful comparisons of risk of serious illness associated with infection by the species of plasmodia. Examining the relative burdens of risk of serious and fatal illness associated with *P. falciparum* and *P. vivax* malaria was the primary objective of this study.

Possible confounding of these findings by co-infections represents an important limitation with this study. Apart from the possibility of cryptic co-infection with *P. falciparum*, which may be very difficult to observe by microscopy, other endemic infectious diseases occurred on Sumba. None of these possibilities was ruled out by laboratory investigation, and some of these agents may have been the primary cause of illness among the patients diagnosed with malaria. Exhaustive laboratory investigations of patients with malaria elsewhere have demonstrated substantial contributions by co-infections or co-morbidities for both *P. falciparum* and *P. vivax*.^20^,^21^

Clinical and laboratory evidence in this study and others suggests that vivax malaria could have been an important contributing determinant of serious illness among patients with that diagnosis. The densities of parasitemia among all classes of illness with vivax malaria were consistently and strikingly lower than those among the patients with falciparum malaria (Figure 6), and a substantially lower risk of serious illness with a diagnosis of vivax malaria appeared (Table 2). Likewise, risk of serious illness showed a sharply age-dependent pattern among patients with a diagnosis of *P. falciparum* but not with *P. vivax* (Figure 3). Collectively, these findings suggest that serious and fatal illness associated with *P. vivax* was not often misdiagnosed *P. falciparum* or a mixed infection dominated by that parasite.

Co-infection by other endemic infectious agents cannot be ruled out among the patients at Karitas Hospital diagnosed with malaria. The proportion of patients receiving presumptive antibiotic therapy with a confirmed diagnosis of falciparum or vivax malaria was just 15% and 28%, respectively, whereas 90% and 92% of patients classified as seriously ill recovered after therapy for that diagnosis. Although spontaneous recovery from less threatening infections cannot be ruled out, most of these recoveries appear attributable to antimalarial therapy. That relationship would point to the plasmodia as the dominant etiologic agent.

Among fatalities, however, death obviously occurred despite such therapy and the likelihood of other endemic infections playing a role in that outcome cannot be ruled out. Studies elsewhere,^22^–^24^ however, evaluated fatal cases for the most common endemic co-infections and found few other causes of death more likely than *P. vivax.* Those fatalities exhibited the same range of syndromes observed among the seriously ill patients with a diagnosis of vivax malaria in West Sumba. The proportion of patients classified as having serious illness and suffering a fatal outcome (11.5% with a diagnosis of *P. falciparum*; 9.0% for *P. vivax*) accorded with similar frequencies in other hospital-based studies (6–10% for *P. falciparum*; 8–11% for *P. vivax*).^6^–^10^

Clinical studies support a relationship between serious illness and risk of death with vivax malaria. Rates of reported death under vivax malaria therapy for neurosyphilis in patients during the 1920s and 1930s show consistency with the data from hospitals in endemic zones today. The therapeutic efficacy of induced malaria for tertiary syphilis, an otherwise fatal disease before antibiotics, was only about 35% and hinged upon the severity and number of malaria paroxysms.^25^ Practitioners were therefore relatively aggressive with malaria therapy, and those patients may be considered to have experienced relatively severe disease.^26^ The 5–10% case fatality rates among patients classified as seriously ill with a diagnosis of vivax malaria among patients in endemic zones today approximates the 5–15% rates observed among many thousands of patients challenged with *P. vivax* for therapy of tertiary syphilis.^27^–^34^

As with death caused by many infectious agents, such extremes of virulence by the parasites that cause malaria may hinge upon complex host genetic, nutritional, immunological, and many other factors, as well as co-infections or other underlying disease states. The study at Karitas Hospital does not confirm *P. vivax* as the sole agent of serious and fatal illness in any patient. Indeed, this may be unlikely with either species of parasite, and the observed relative weights of association with serious and fatal illness may encourage and guide more detailed prospective analyses of this question.

A diagnosis of falciparum malaria carried a higher risk of serious illness among hospitalized patients (OR = 2.9; 95% CI = 2.4–3.5), but vivax malaria was nonetheless associated with 32% of that burden. Risk of death among patients with falciparum malaria and classified as having serious illness was indistinguishable from patients with a diagnosis of vivax malaria (OR = 1.3; 95% CI = 0.7–2.5). These findings accorded with a similar retrospective study from Jayapura, Papua; OR = 2.8; 95% CI: 2.0–3.0 for severe disease with falciparum versus vivax malaria, and OR = 0.9; 95% CI: 0.4–2.1 for risk of death with those diagnoses.^6^ In a 4-year prospective hospital-based study at Timika, Papua in eastern Indonesia,^7^ admitted patients were slightly less likely to be classified as having severe disease with a diagnosis of falciparum versus vivax malaria (OR = 0.8; 95% CI: 0.8–0.9), but equally likely to suffer death with that classification (OR = 1.3; 95% CI: 0.9–1.8). The rates of severe and fatal falciparum malaria among the seriously ill at Timika (20% and 11%) generally accorded with those observed at Karitas hospital (26% and 12%), as did those with vivax malaria; 23% and 7% at Timika, and 11% and 9% at West Sumba. Unlike the study at Timika,^7^ young children at Karitas hospital were not strikingly more susceptible to serious illness associated with vivax malaria, even though such a pattern was clearly evident with falciparum malaria (Figure 3). Further surveys of malaria morbidity and mortality at other hospitals in the region are required to establish such frequencies as general trends in eastern Indonesian endemic zones.

The spectrum of syndromes among seriously ill patients with vivax or falciparum malaria resembled those observed in the prospective hospital-based study from Timika, Papua in eastern Indonesia, and in the Sepik region of Papua New Guinea^7^,^8^; severe anemia dominated and altered mental state syndromes figured prominently in the study. Records of most patients at Sumba did not include thrombocyte counts, but hemorrhage was documented in some patients with vivax malaria. Sometimes severe thrombocytopenia is a well known and prominent, if not dominant, feature of acute vivax malaria in hospitalized patients.^35^ The relatively lower frequencies of multiple organ involvement in this study may reflect the relative insensitivity of the classifications as a consequence of the few laboratory assessments available at Karitas Hospital.

In summary, this retrospective study of the 24-month malaria experience of a single hospital in eastern Indonesia suggests *P. vivax*, along with myriad possible co-determinants, contributes substantially to the burdens of hospitalizations, serious illness, and death associated with the malaria endemic to this community. Although risk of serious illness with a diagnosis of vivax malaria was significantly less than with that of falciparum malaria, risk of death among patients with serious disease was approximately equal between those diagnoses. Infants, children, and adolescents bore most of the burdens of serious and fatal illness associated with falciparum malaria, whereas all age groups appeared vulnerable to serious and fatal illness associated with vivax malaria. Despite the perception of vivax malaria as a benign infection, this parasite may be contributing to or causing serious and fatal illness in eastern Indonesia.

## Figures and Tables

**Table 1:** Morbidity and mortality associated with falciparum and vivax malaria during 2008 and 2009 at Karitas Hospital, West Sumba*

Demographic groups	Admissions			Serious disease (% of admissions)			Death (% of serious)		
	*PF*	*PV*	Mix	*PF*	*PV*	Mix	*PF*	*PV*	Mix
Infants (< 12 months)	64	198	5	30 (47)	19 (10)	1 (20)	3 (10)	3 (16)	0 (0)
Children (≥ 12 months and < 10 years)	658	557	31	264 (40)	75 (14)	7 (23)	33 (13)	6 (8)	1 (14)
Adolescents (≥ 10 and < 15 years)	150	149	9	33 (22)	13 (9)	4 (44)	5 (15)	0 (0)	0 (0)
Adults (≥ 15 and < 60 years)	633	808	23	70 (11)	70 (9)	2 (9)	5 (7)	7 (10)	0 (0)
Men	310	368	11	28 (9)	24 (7)	2 (18)	3 (11)	2 (8)	0 (0)
Women	323	440	12	42 (13)	46 (10)	0 (0)	2 (5)	5 (11)	0 (0)
Not pregnant	234	389	11	30 (13)	37 (10)	0 (0)	1 (3)	4 (11)	0 (0)
Pregnant	89	51	1	12 (14)	9 (18)	0 (0)	1 (8)	1 (11)	0 (0)
Elderly (≥ 60 years)	36	125	3	3 (8)	22 (18)	1 (33)	0 (0)	2 (9)	1 (100)
Total	1,541	1,837	71	400 (26)	199 (11)	15 (21)	46 (12)	18 (9)	2 (13)

* *PF* = *Plasmodium falciparum*; *PV* = *Plasmodium vivax.*

**Table 2:** Odds of serious illness associated with falciparum versus vivax malaria among groups*

All age groups	Serious		Infants (< 12 months)	Serious	
	Yes	No		Yes	No
*Plasmodium falciparum*	400	1,141	*P. falciparum*	30	34
*Plasmodium vivax*	199	1,638	*P. vivax*	19	179
OR (95% CI)	2.9 (2.4–3.5)		OR (95% CI)	8.3 (4.0–17.4)	
Children (≥ 12 months and < 10 years)	Serious		Adolescents (≥ 10 and < 15 years)	Serious	
	Yes	No		Yes	No
*P. falciparum*	264	394	*P. falciparum*	33	117
*P. vivax*	75	482	*P. vivax*	13	136
OR (95% CI)	4.3 (3.2–5.8)		OR (95% CI)	3.0 (1.4–6.4)	
Adults (≥ 15 and < 60 years)	Serious		Elderly (≥ 60 years)	Serious	
	Yes	No		Yes	No
*P. falciparum*	70	563	*P. falciparum*	3	33
*P. vivax*	70	738	*P. vivax*	22	103
OR (95% CI)	1.3 (0.9–1.9)		OR (95% CI)	0.4 (0.1–1.6)	
Pregnant	Serious		Not pregnant	Serious	
	Yes	No		Yes	No
*P. falciparum*	12	77	*P. falciparum*	30	204
*P. vivax*	9	42	*P. vivax*	37	352
OR (95% CI)	0.7 (0.3–2.1)		OR (95% CI)	1.4 (0.8–2.4)	

* OR = odds ratio; CI = confidence interval.

**Table 3:** Odds of fatal outcome with serious illness with a diagnosis of falciparum versus vivax malaria*

All age groups	Fatal	
	Yes	No
*Plasmodium falciparum*	46	354
*Plasmodium vivax*	18	181
OR (95% CI)	1.3 (0.7–2.5)	

* OR = odds ratio; CI = confidence interval.

**Table 4:** Distribution of multiple serious and fatal malaria syndromes*

Syndromes (per patient)	Serious			Fatal		
	*PF*	*PV*	Mix	*PF*	*PV*	Mix
1	359	191	13	36	16	2
2	40	8	2	10	2	0
3	1	0		0	0	0
Total	400	199	15	46	18	2

* *PF* = *Plasmodium falciparum*; *PV* = *Plasmodium vivax.*

## References

[1] Kitchen SF, Boyd MF (1949). Vivax malaria. Malariology.

[2] Price RN, Douglas NM, Anstey NM (2009). New developments in *Plasmodium vivax* malaria: severe disease and the rise of chloroquine resistance. Curr Op Infect Dis.

[3] Guerra CA, Howes RE, Patil AP, Gething PW, Van Boekel TP, Temperly WH, Kabaria CW, Tatem AJ, Manh BH, Elyazar IR, Baird JK, Snow SI (2010). The international limits and population at risk of *Plasmodium vivax* transmission in 2009. PLoS NTD.

[4] Hay SI, Guerra CA, Gething PW, Patil AP, Tatem AJ, Noor AM, Kabaria CW, Manh BH, Elyazar IR, Brooker S, Smith DL, Moyeed RA, Snow RW (2009). A world malaria map: *Plasmodium falciparum* endemicity in 2007. PLoS Med.

[5] Dhingra N, Jha P, Sharma VP, Cohen AA, Jotkar RM, Rodriguez PS, Bassani DG, Suraweera W, Laxminarayan R, Peto R, Collaborators Million Death Study (2010). Adult and child malaria mortality in India: a nationally representative mortality survey. Lancet.

[6] Barcus MJ, Basri H, Picarima H, Manyakori C, Sekartuti, Elyazar I, Bangs MJ, Maguire JD, Baird JK (2007). Demographic risk factors for severe and fatal vivax and falciparum malaria among hospital admissions in northeastern Papua, Indonesia. Am J Trop Med Hyg.

[7] Tjitra E, Anstey NM, Sugiarto P, Warikar N, Kenangalem E, Karyana M, Lampah DA, Price RN (2008). Multi-drug resistant *Plasmodium vivax* associated with severe and fatal malaria: a prospective study in Papua, Indonesia. PLoS Med.

[8] Genton B, D’Acremont V, Rare L, Baea K, Reeder JC, Alpers MP, Muller I (2008). *Plasmodium vivax* and mixed infections are associated with severe disease with severe malaria in children: a prospective cohort study from Papua New Guinea. PLoS Med.

[9] Kochar DK, Tanwar GS, Khatri PC, Kochar SK, Senegar GS, Gupta A, Kochar A, Middha S, Acharya J, Sexena V, Pakalapati D, Garg S, Das A (2010). Clinical features of children hospitalized with malaria—a study from Bikaner, northwest India. Am J Trop Med Hyg.

[10] Sharma A, Khanduri U (2009). How benign is benign tertian malaria?. J Vector Borne Dis.

[11] Oh M, Shin H, Shin D, Kim U, Lee S, Kim N, Choi M, Chai J, Choe K (2001). Clinical features of vivax malaria. Am J Trop Med Hyg.

[12] Beg MA, Sani N, Mehraj V, Jafri W, Khan MA, Malik A, Menezes E, Hussain R, Smego R (2008). Comparative features and outcomes of malaria at a tertiary care hospital in Karachi, Pakistan. Int J Infec Dis.

[13] Rodrigues-Morales AJ, Ferrer MV, Barrera MA, Pacheco M, Daza V, Franco-Paredes C (2009). Imported cases of malaria admitted to two hospitals of Margarita Island, Venezuela, 1998–2005. Travel Med Infect Dis.

[14] Koh KH, Chew PH, Kiyu A (2004). A retrospective study of malaria infections in an intensive care unit of a general hospital in Malaysia. Singapore Med J.

[15] Elyazar IR, Hay SI, Baird JK (2011). Malaria distribution, prevalence, drug resistance, and control in Indonesia. Adv Parasitol.

[16] Elyazar IR, Gething PW, Patil AP, Rogayah H, Kusriastuti R, Wismarini DM, Tarmizi SN, Baird JK, Hay SI (2010). *Plasmodium falciparum* malaria endemicity in Indonesia in 2010. PLoS One 6.

[17] Maguire JD, Lederman ER, Barcus MJ, O’meara WA, Jordan RG, Duong S, Muth S, Sismadi P, Bangs MJ, Prescott WR, Baird JK, Wongsrichanalai C (2006). Production and validation of durable, high quality standardized malaria microscopy slides for teaching, testing and quality assurance during an era of declining diagnostic proficiency. Malar J.

[18] Syafruddin D, Krisin, Asih P, Sekartuti, Dewi RM, Coutrier F, Rozy IE, Susanti AI, Elyazar IR, Sutamihardja A, Rahmat A, Kinzer M, Rogers WO (2009). Seasonal prevalence of malaria in West Sumba district, Indonesia. Malar J.

[19] Greenwood BM, Armstrong JR (1991). Comparison of two simple methods for determining malaria parasite density. Trans R Soc Trop Med Hyg.

[20] Taylor TE, Fu WJ, Carr RA, Whitten RO, Mueller JS, Fosiko NG, Lewallen NG, Molyneux ME (2004). Differentiating the pathologies of cerebral malaria by postmortem parasite counts. Nat Med.

[21] Lampah DA, Yeo TW, Hardianto SO, Tjitra E, Kenangalem E, Sugiarto P, Price RN, Anstey NM (2011). Coma associated with microscopy-diagnosed *Plasmodium vivax*: a prospective study in Papua, Indonesia. PLoS Negl Trop Dis.

[22] Valecha N, Pinto RG, Turner GD, Kumar A, Rodrigues S, Dubhashi NG, Rodrigues E, Banaulikar SS, Singh R, Dash AP, Baird JK (2009). Histopathology of fatal respiratory distress caused by *Plasmodium vivax* malaria. Am J Trop Med Hyg.

[23] Habib AG, Singh KS (2004). Respiratory distress among nonimmune adults with imported malaria. Infection.

[24] Andrade BB, Reis-Filho A, Souza-Neto SM, Clarêncio J, Camargo LM, Barral A, Barral-Netto M (2010). Severe *Plasmodium vivax* malaria exhibits marked inflammatory imbalance. Malar J.

[25] Nicol WD (1932). A review of seven years malaria therapy in general paralysis. J Ment Sci.

[26] Nicol WD (1927). The care and management of induced malaria. J Ment Sci.

[27] Eldridge WW, Lind JE, Silk SA, Trentzsch PJ (1925). Treatment of paresis: results of inoculation with the organism of benign tertian malaria. JAMA.

[28] Ferraro A, Fong TC (1927). The malaria treatment of general paresis. J Nerv Ment Dis.

[29] Freeman W, Eldridge WW, Hall RC (1934). Malaria treatment of dementia paralytica: results in 205 cases after five to eleven years. South Med J.

[30] O’Leary PA, Welsh AL (1933). Treatment of neurosyphilis with malaria: observations on nine hundred and eighty-four cases in the last nine years. JAMA.

[31] Paulian D (1935). Statistic misconception about six years of malaria therapy. Rev Neurol.

[32] Fong TC (1937). A study of the mortality rate and complications following therapeutic malaria. South Med J.

[33] Krauss W (1932). Analysis of reports of 8,354 cases of IMPF-Malaria. South Med J.

[34] James SP, Nicol WD, Shute PG (1936). Clinical and parasitological observations on induced malaria. Proc R Soc Med.

[35] Rodriguez-Morales AJ, Sanchez E, Vargas M, Picolo C, Colina R, Arria M (2006). Anemia and thrombocytopenia in children with *Plasmodium vivax*. J Trop Pediatr.

